# Supplementary material for: Unconventional superconductivity without doping in infinite-layer nickelates under pressure
Source: Nat Commun. 2024 May 10;15:3952. doi: 10.1038/s41467-024-48169-5 (PMC11087552; doi:10.1038/s41467-024-48169-5)
Supplement: Supplementary file 1 — Supplementary Information [file 41467_2024_48169_MOESM1_ESM.pdf]

Supplementary Information:
Unconventional superconductivity without doping in infinite-layer nickelates under pressure

Simone Di Cataldo,[1,*] Paul Worm,[1] Jan M. Tomczak,[1,2] Liang Si,[3] and Karsten Held[1]

[1]*Institut für Festkörperphysik, Technische Universität Wien, 1040 Wien, Austria*
[2]*King's College London, London, WC2R 2LS, United Kingdom*
[3]*School of Physics, Northwest University, Xi'an 710127, China*

(Dated: March 26, 2024)

This Supplemental information contains additional information on the s employed in Section I and additional results in Sections II. Specifically, Section I A provides an overview; Section I B gives details of the density functional theory (DFT) calculation; Section I C gives details of the structural relaxation; Section I D gives details on the Wannierization of the DFT bands at the different pressures. Section I E provides information on the constrained random phase approximation (cRPA) calculation of the interaction parameters; Section I F gives further details on the dynamical mean-field theory (DMFT) calculations; and Section I G on the dynamical vertex approximation (DΓA). Additional results are presented in Section II. This includes DFT+DMFT calculations for the 10- and 1-band model in Section II A, a comparison of the DΓA T_c to that of $Sr_xNd_{1-x}NiO_2$ in Section II B, and an in-depth test of the virtual crystal approximation (VCA) in Section II C. Finally, Section II D addresses how the possible limitations of the methods employed should not impact the main message of this paper. In particular, we address the absence of self-consistency between the DFT and DMFT calculations, the possibility of a temperature dependence of the occupation of the Ni $d_{x^2-y^2}$ orbital, and a possible competition of superconductivity with an antiferromagnetic phase.

I. METHODOLOGICAL DETAILS

In this section, we provide a more detailed description of the methods employed in the main text. The interested reader can find the input files for the whole set of calculations in the associated data repository [1].

A. Complete workflow

We start with presenting a flowchart of the entire methodology as an overview in Fig. S1; and further details on the individual steps can then be found in the subsequent Sections. We start by computing the in-plane lattice parameter as a function of pressure from the STO equation of state in DFT and fix the PNO in-plane lattice parameters to that of the substrate. Next, we determine the out-of-plane lattice parameter of PNO, as detailed in I C. From the relaxed crystal structure, we compute the electronic dispersion $\epsilon_{\mathbf{k}}$ using Density Functional Theory and extract the Wannier functions for 10 bands (Ni-d + Pr-d) and 1 band (Ni-$d_{x^2-y^2}$).

As described in Ref. [2], we first perform a DMFT calculation for the 10 bands case, which allows us to obtain the filling of the Ni-$d_{x^2-y^2}$ in the case of interacting Ni and Nd orbitals. The values of interaction employed in this calculation are reported in Table S3. Using the thus computed filling of the Ni-$d_{x^2-y^2}$ band, we perform a second DMFT calculation for the single Ni-$d_{x^2-y^2}$ band, from which we obtain the local two-particle Green's function $G^{(2)}(i\nu, i\nu', i\omega)$.

Finally, using ladder-DΓA we compute the non-local vertex and the (magnetic) susceptibility at various temperature. The superconducting T_c is then estimated as the temperature for which the leading eigenvalue of the linearized Eliashberg equation is equal to one, as in Ref. [2]. The source code of the used ladder-DΓA code is available in Ref. [3].

B. Density Functional Theory

DFT calculations are performed with the Vienna ab-initio Simulation Package (VASP) [4], using the Perdew-Burke-Ernzerhof adapted for solids (PBESol) [5] exchange-correlation functional. We employ the projector-augmented waves (PAW) pseudopotentials provided within the VASP package [6], for which praseodymium is constructed with f orbitals frozen within the core states. Integration over the Brillouin zone was performed over a uniformly-spaced grid with a spacing of 0.25 Å^{-1}, and a Gaussian smearing of 0.05 eV. The input and output files for all calculations performed are available as additional data [1].

* simone.dicataldo@uniroma1.it

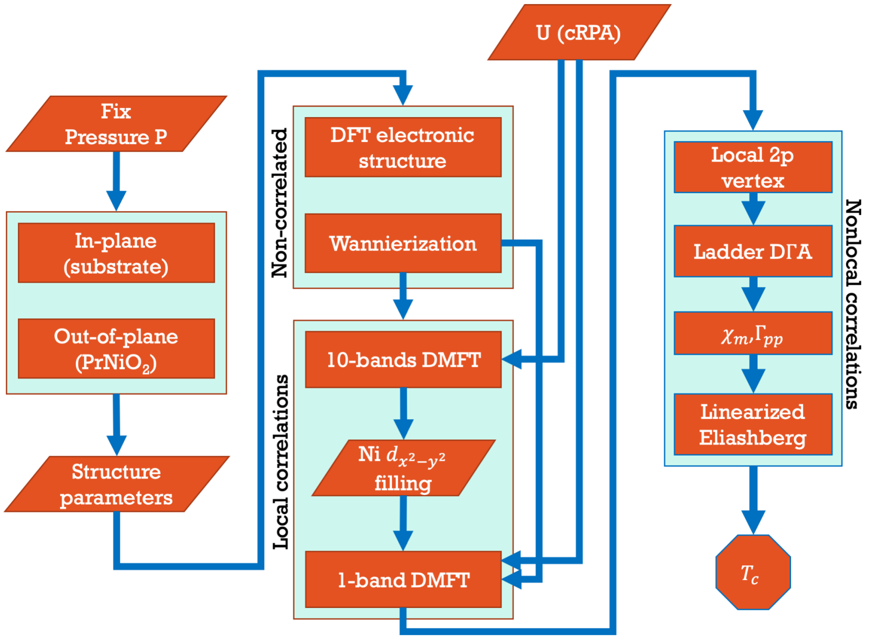

Figure S1: Flowchart summarizing the whole workflow employed in the project.

At the DFT level, the Sr-doping of the $PrNiO_2$ crystal was simulated by means of the virtual crystal approximation (VCA) to simulate a virtual $Pr_{1-x}Sr_x$ atom following the method describe by Bellaiche and Vanderbilt [7], and implemented in VASP. The use of this approximation, despite the different core of Sr and Pr is justified by the fact that in the system studied both, Pr and Sr, act mostly as charge donors and spacers between NiO_2 planes, and do not contribute significantly to the states at the Fermi energy. Nevertheless, we checked extensively the quality of the VCA against Vergard's law, and on the structural and electronic properties of $Pr_{0.75}Sr_{0.25}NiO_2$, compared with the results on a 2×2×2 supercell. As these checks are quite extensive and we do not want to interrupt the further discussion of the workflow here, we present in Section II C, Figures S16 (Vergard's law) and S17 (comparison with 2×2×2 supercell). This aspect of Figure S4, which we present already in Section I C for its information on how to obtain the *c*-axis parameter, is also discussed in Section II C. All these tests confirmed that the VCA is consistent with the results obtained for supercells in the structure studied.

C. Structural Relaxation

In this section, we report the main results for the structural relaxation of the $SrTiO_3$ (STO) substrate. In Fig. S2 we show the equation of states along with the results from a fit with the Birch-Murnaghan equation for STO as a function of pressure. In Fig. S3 we show the corresponding lattice parameter.

The effect of isotropic pressure was computed in two steps, including the effect of the $SrTiO_3$ (STO) substrate on the in-plane lattice parameter, as well as the effect of pressure on the c axis. This strategy differs substantially from Ref. [8], where the in-plane lattice constant is kept constant, as it is not suited to study the rather isotropic pressures that are realized in experiments using diamond anvil cells.

Our method of computing the crystal structure of $Pr_{1-x}Sr_xNiO_2$, on the other hand, is motivated by the consideration of how the actual crystal is grown in experiments. Infinite-layer nickelates are grown over a perovskite substrate. Often STO is used [9–13], this also includes the experiments by Wang *et al.* [14] which motivated the present study. But, $NdGaO_3$ (NGO) [15] and $(LaAlO_3)_{0.3}(Sr_2TaAlO_6)_{0.7}$ (LSAT) [16] was employed as well. The nickelate layer is grown with a thickness between 10 and 100 nm [9–13, 15, 17] over a bulk substrate which can be regarded as infinite, and capped again with a few layers of the same substrate. Hence we work in the hypotheses that:

1. The nickelate is forced to assume the same in-plane lattice constant of the substrate on which it is grown.
2. In the xy plane the elastic response of the system is dominated by the substrate, due to the substrate being much thicker.

3. Along the z direction, the nickelate is not constrained by the substrate, hence its response is independent from it.

The above is then modelled in the two following steps:

1. We compute the equation of state for bulk STO in the cubic phase. At a given pressure, the equation of state is used to extract the in-plane lattice constants $a = b$ (Supplementary Figure S2).

2. With a and b fixed to the value given by STO at the chosen pressure, we compute the enthalpy of the nickelate phase as a function of the c axis, see Fig. S4. The c value that minimizes the enthalpy corresponds to the equilibrium value.

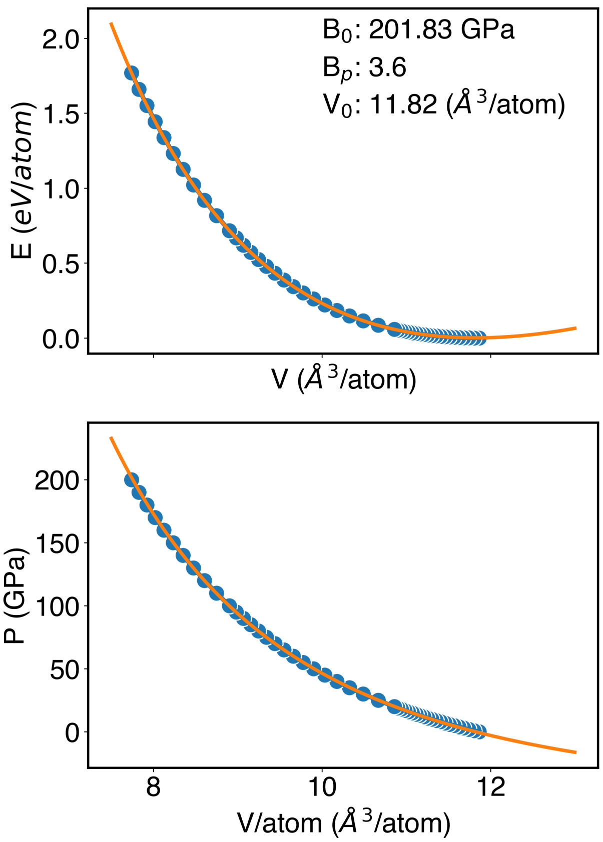

Figure S2: Equations of state $E(V)$ and $P(V)$ for $SrTiO_3$ from 0 to 200 GPa. The blue dots and the orange line denote the values computed within Density Functional Theory and the fit, using the Birch-Murnaghan equation of state [18]. In addition, we report the results of the curve fit.

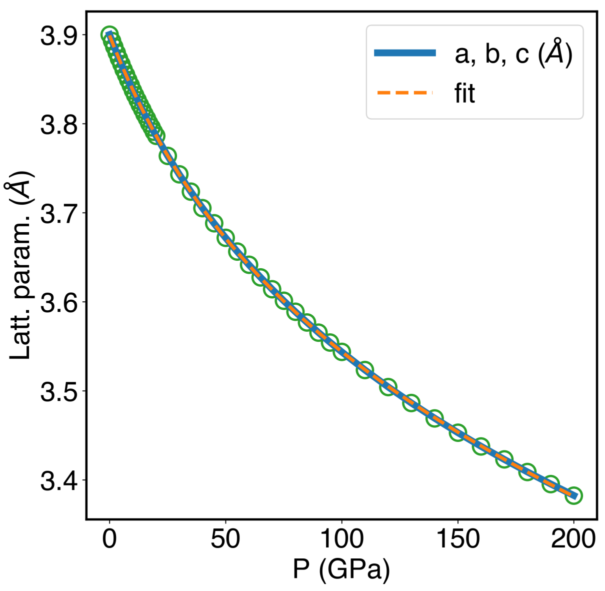

Figure S3: Lattice parameter of STO as a function of pressure from our DFT calculations.

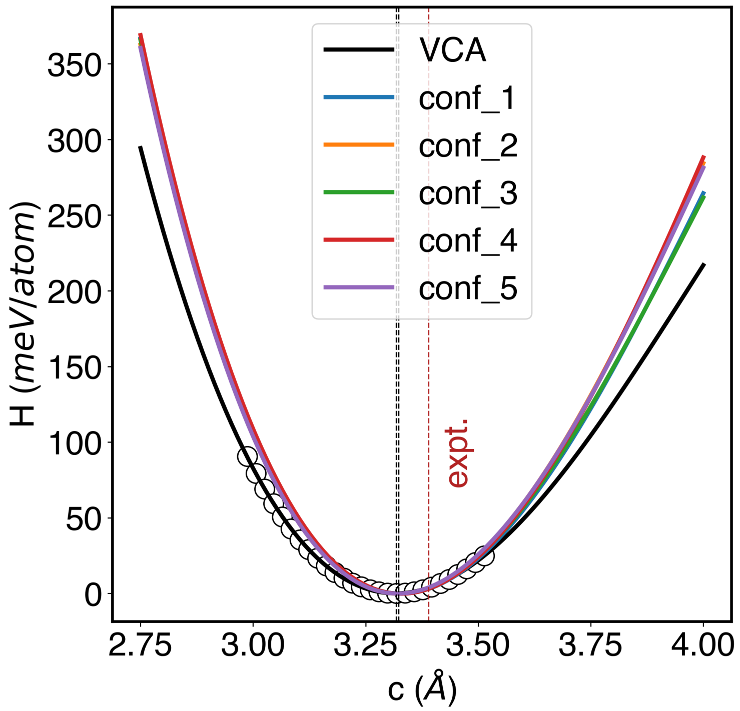

Figure S4: Enthalpy versus value of the c axis for $Pr_{0.75}Sr_{0.25}NiO_2$ using the VCA and in five different supercells. The VCA value is shown as a black line, and individual points are shown as black dots. The results for the supercells are shown as colored lines.

D. Wannier Functions

The DFT bandstructure calculated in VASP is subsequently mapped onto a 10- and a 1-band Wannier basis using maximally localized Wannier orbitals and the `wannier90` code [19]. Here, d orbitals centered on Pr and Ni were used for the initial projections for the 10-bands calculations, and a single $d_{x^2-y^2}$ orbital centered on Ni was used as initial projection for the 1-band calculation. The reader interested in the energy ranges for the disentanglement and frozen windows of each calculation can find

the corresponding input files in [1]. The Hamiltonian $H(\mathbf{k})$ for the 10-band calculations was obtained as the Fourier transform of the `wannier90` output. The Hamiltonian for the 1-band calculation was obtained by extracting the hoppings t, t', t'' from the Wannier Hamiltonian, and only then performing the Fourier transform. That is, the hopping parameters have been cut off at next-next-nearest neighbors.

Fig. S5 shows the direct comparison between Wannier and DFT bands. While there are some deviations of the 10-band model and the DFT band above the Fermi energy where additional bands –besides the 10-bands considered– cross, the agreement at low energies is excellent. And this is the relevant region for the subsequent DMFT calculation.

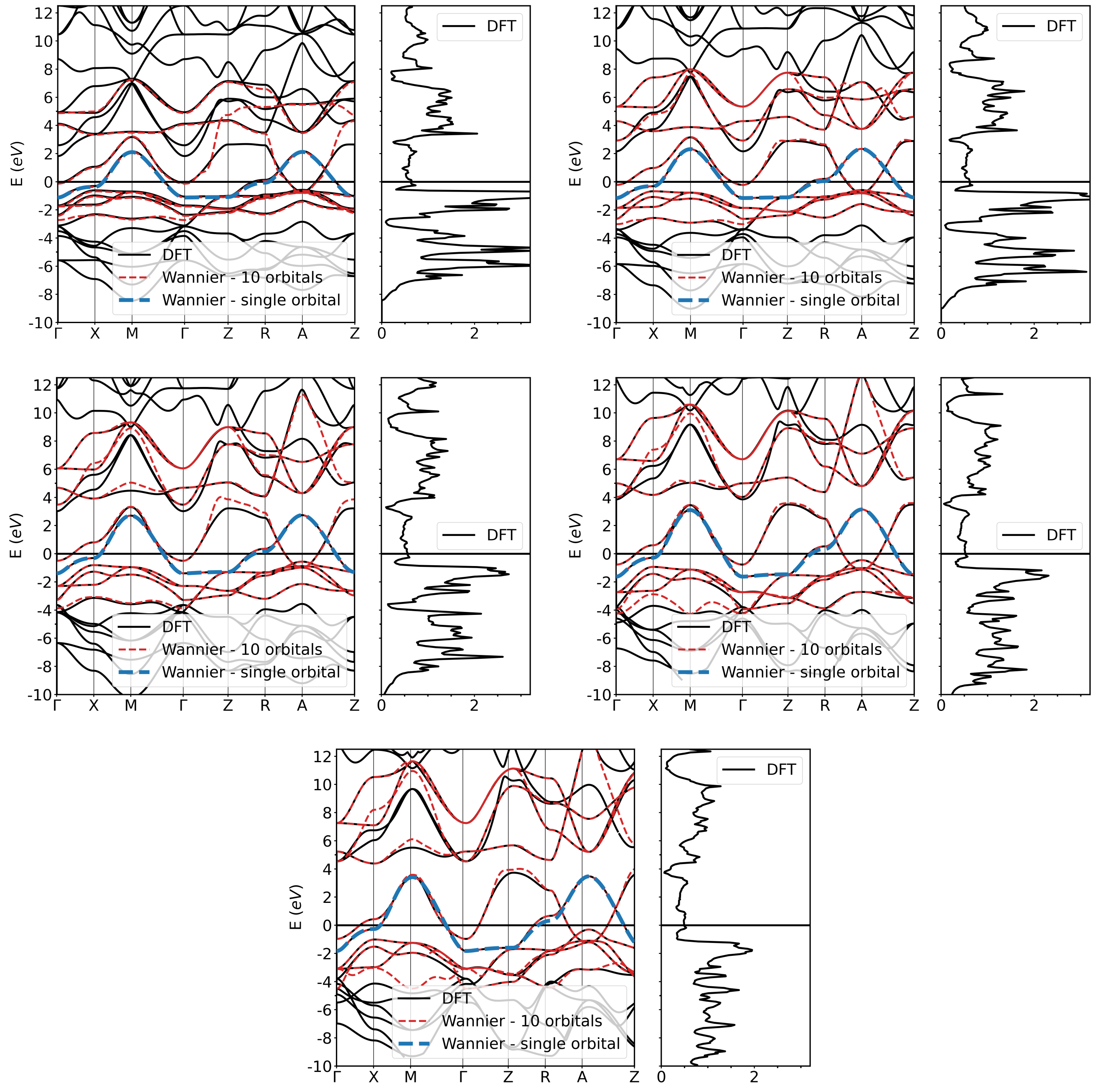

Figure S5: DFT electronic band structure and 10- and 1- band wannierization as a function of pressure. The DFT bands, the 10 bands wannierization and the 1 band wannierization are shown as black solid, red dashed, and blue dashed lines, respectively. The figures are shown in order of ascending pressure. From top left to bottom right: 0, 12, 50, 100, and 150 GPa, respectively.

From the wannierization, we can also extract the hopping amplitudes t, t', and t'' for nearest, next-nearest, and next-next-nearest neighbour hopping for the effective 1-orbital model. These parameters for different pressures and dopings are reported

in Table S1 and serve as a DMFT input for the 1-band calculation. In case of the fully-fledged 10-band DMFT calculation, we used the full $H(\mathbf{k})$ of the Wannier bands, without restriction to shorter-range hoppings.

Pressure (GPa)	Doping	t (eV)	t' (eV)	t'' (eV)	U (eV)	U (t)	n_{eff} Ni $d_{x^2-y^2}$ ($e/band$)
0	0%	-0.388	0.097	-0.049	3.4	8.77	0.973
12	0%	-0.416	0.099	-0.052	3.4	8.17	0.965
50	0%	-0.483	0.109	-0.059	3.4	7.04	0.933
100	0%	-0.558	0.112	-0.067	3.4	6.10	0.887
150	0%	-0.617	0.118	-0.073	3.4	5.51	0.858
0	18%	-0.388	0.097	-0.049	3.4	8.77	0.838
12	18%	-0.416	0.099	-0.052	3.4	8.17	0.831
50	18%	-0.483	0.109	-0.059	3.4	7.04	0.817
100	18%	-0.558	0.112	-0.067	3.4	6.10	0.775
150	18%	-0.617	0.118	-0.073	3.4	5.51	0.742
0	0%	-0.388	0.097	-0.049	3.4	8.77	0.973
0	10%	-0.388	0.097	-0.049	3.4	8.77	0.902
0	15%	-0.388	0.097	-0.049	3.4	8.77	0.862
0	20%	-0.388	0.097	-0.049	3.4	8.77	0.823
0	25%	-0.388	0.097	-0.049	3.4	8.77	0.790
0	30%	-0.388	0.097	-0.049	3.4	8.77	0.767
50	0%	-0.483	0.109	-0.059	3.4	7.04	0.933
50	5%	-0.483	0.109	-0.059	3.4	7.04	0.905
50	10%	-0.483	0.109	-0.059	3.4	7.04	0.872
50	15%	-0.483	0.109	-0.059	3.4	7.04	0.840
50	20%	-0.483	0.109	-0.059	3.4	7.04	0.802
50	25%	-0.483	0.109	-0.059	3.4	7.04	0.762
50	30%	-0.483	0.109	-0.059	3.4	7.04	0.722
50	40%	-0.483	0.109	-0.059	3.4	7.04	0.643

Table S1: Calculated quantities for the single-band Hubbard model of the Ni $d_{x^2-y^2}$ band at the pressures and physical Sr-doping values x employed in this paper. The effective filling $n = 1 - \delta$ is reported.

E. Constrained random phase approximation

This Wannier Hamiltonian needs to be supplemented by the Coulomb interaction. To this end, the static Hubbard interaction U was computed from first principles using the constrained random phase approximation (cRPA) in the Wannier basis [20] for entangled band-structures [21]. Here, the underlying electronic structure was computed from DFT using a full-potential linearized muffin-tin orbital (fplmto) method [22] and the local density approximation, applied to bulk $LaNiO_2$ using the relaxed tetragonal structures from Sec. I C. The calculations use 10^3 reducible $\mathbf{k}$-points, and a muffin-tin radius (RMT) for Ni of 1.97 Bohr radii, except for P = 100 GPa, where RMT=1.9. At P = 50 GPa the difference in RMT changes U by merely 0.4%. The cRPA results for the 1-band model are shown in Table S2. Note that the Hubbard U can indeed display a non-trivial dependence on pressure [23]. E.g., in cuprates in-plane compression increases the local interaction in a $d_{x^2-y^2}$ setting [24].

We find both the screened and the bare interaction—U and V—to be essentially insensitive to pressure: Both the localization of the $d_{x^2-y^2}$-derived Wannier function and the screening remain constant. This justifies keeping U unchanged with pressure. To account for the frequency-dependence of U in cRPA a slightly larger value is needed, and we use a fixed U = 3.4 eV as is stated in Table S1 was already employed in [2].

The disparity between a considerable change of the hopping elements but only very minute changes of U can be rationalized as follows: Under pressure the Ni atoms are pushed closer together. Thus the overlap between orbitals *on neighboring sites* which enters in the hopping amplitudes increases. Instead the local Coulomb interaction involves an integral over four Wannier functions *on the same* atomic site. As the shape and Wannier spread of the orbitals hardly changes at the considered pressures, U is much more insensitive to pressure.

Given their weak pressure dependence, we also keep the interaction parameters fixed for the 10-band calculation. These have been calculated before in cRPA [25] and are displayed in Table S3. In [25] the interaction matrices for the 5 Ni and 5 La(Pr) d-orbitals were calculated in VASP within GGA in the framework of Perdew-Burke-Ernzerhof for solids version (GGA-PS) on a k-mesh of 13×13×15, using the static limit U=$U(\omega$=0) and U-J average according to [26].

For infinite-layer nickelates with an electronic configuration close to $3d^9$, the relevant Coulomb interaction is between the e_g states, which is of Kanamori type. Note that the Ni t_{2g} orbitals can, for all practical purposes, considered to be filled, see Fig. S6.

P [GPa]	0	12.1	20	50	100
U [ev]	2.88	2.88	2.88	2.96	2.93
V [eV]	19.4	19.3	19.2	19.3	19.0

Table S2: Local Hubbard interaction U and bare (unscreened) Coulomb interaction V in the maximally localized Wannier function basis for the 1-band (Ni-$d_{x^2-y^2}$-band) model as calculated by cRPA.

For tetragonal symmetry this e_g-e_g interaction must have the Kanamori form, which justifies the use of the simplified Kanamori interaction instead of the full Slater one.

Pressure (GPa)	Temperature (K)	U$_{Ni}$ (eV)	J$_{Ni}$ (eV)	U'$_{Ni}$ (eV)	U$_{Nd}$ (eV)	J$_{Nd}$ (eV)	U'$_{Nd}$ (eV)
0	300	4.40	0.65	3.10	2.50	0.25	2.00
12	300	4.40	0.65	3.10	2.50	0.25	2.00
50	300	4.40	0.65	3.10	2.50	0.25	2.00
100	300	4.40	0.65	3.10	2.50	0.25	2.00
150	300	4.40	0.65	3.10	2.50	0.25	2.00

Table S3: Intra- and inter-orbital Coulomb repulsion (U, U') and Hund coupling J for the Kanamori Hamiltonian employed in the 10-band DMFT calculations, taken from Ref. [25].

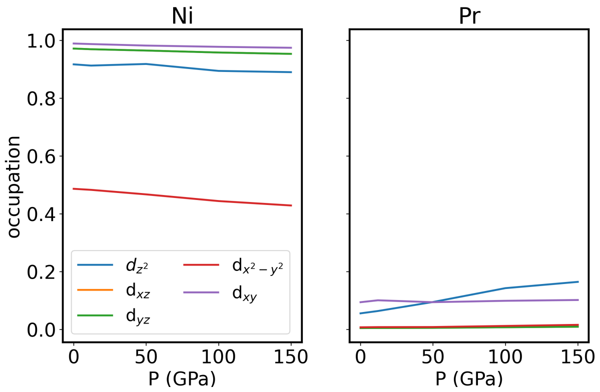

Figure S6: Occupation of the Ni (left) and Pr (right) d-orbitals under pressure at $x = 0$.

F. Dynamical mean-field theory

DMFT[27, 28] calculations were performed using `w2dynamics` version 1.1.3 [29, 30]. All the input files are available as extended data at Ref. [1].

a. 10-band case In the 10-band case, we employ a Kanamori Hamiltonian, and considered the Nd and Ni atoms as two different impurity sites, with interactions described in Supplementary Table S3. The convergence of the local Green's function was achieved through a three-step process. The first two steps were performed with an increasing sampling of the quantum Monte-Carlo solver, for a total of 30 iterations, while a third, final step with a larger number of iterations was employed to better sample the Green's function. We did not feed back the changed DMFT charge into the DFT Kohn-Sham potential, as done in charge self-consistent DFT+DMFT, because the changes of occupations were minor.

b. 1-band case In the 1-band case we employed a Hamiltonian with only density-density interaction and the same two-step scheme of the 10-band case, with the addition of a fourth step with much larger sampling to obtain the local two-particle Green's function. Note: the Hamiltonian was obtained from the Wannierization of the Ni-$d_{x^2-y^2}$ band, in which all values but t, t', t'' were set to zero. For further details see Sect. I D.

G. Dynamical vertex approximation

Based on this 1-band model, we perform ladder DΓA calculations to obtain the nonlocal magnetic and superconducting susceptibility starting from the local two-particle Green's function. As explained in more details in [31], from the local two-particle Green's function, first a local vertex Γ that is irreducible in the particle-hole channel is determined. From the local Γ in turn we calculate the DΓA lattice susceptibility using the Moriya-λ correction [32–35]. In our case, the dominant susceptibility is the magnetic one. The accuracy of this treatment has been convincingly demonstrated in [36] by comparing it with numerical approaches such as diagrammatic quantum Monte Carlo. Using self-consistent DΓA instead of the Moriya-λ correction gives comparable results [37] for the antiferromagentic susceptibility.

From this susceptibility in turn we extract the irreducible vertex in the particle-particle or Cooper channel Γ_{pp}, cf. [31, 38]. For making the approach numerically feasible at the considered temperatures, we do not feed back the particle-particle vertex to the particle-hole channel as in the fully fledged parquet approach [39]. Note that the particle-particle contributions only becomes large in the immediate vicinity of the superconducting phase transition. Hence, outside the realms of Kosterlitz-Thouless physics we only expect a minor modification of T_c.

Finally, from Γ_{pp} and the bare susceptibility, we obtain the superconducting eigenvalue λ. Superconductivity is signalled by the leading eigenvalue (in our case this is in the d-wave channel) approaching one.

Following Ref. [2], where low critical temperatures did not allow for a direct calculation below the critical temperature, the superconducting eigenvalue λ was fitted with the function $\lambda(\beta) = A - B * log(\beta)$ to extrapolate the result to $\lambda = 1$. Here β is the inverse temperature, and A and B are fit parameters. In Fig. S15 we show the superconducting eigenvalue calculated as function of pressure and temperature along with the fitted extrapolating function. In particular, we show that even excluding the two points at the lowest temperature, the extrapolated value is within up to 20% of the true value. Note that comparing these two extrapolations rather gives an upper estimate of the error associated with the extrapolation $\lambda \to 1$.

II. ADDITIONAL RESULTS

In this section, we report additional results from our study.

A. DFT+DMFT

In Fig. S7 we show the effective filling of the Ni-$d_{x^2-y^2}$ band, obtained from the 10-band DMFT calculations as described in the main text. We computed the doping dependency at 0 and 50 GPa, and the pressure dependence at fixed Sr concentration $x = 0.18$. In general, the filling of the Ni-$d_{x^2-y^2}$ band decreases linearly with increasing pressure and with larger Sr concentration. However, at 0 GPa the decrease flattens out for $x \geq 0.3$, while it remains linear at 50 GPa.

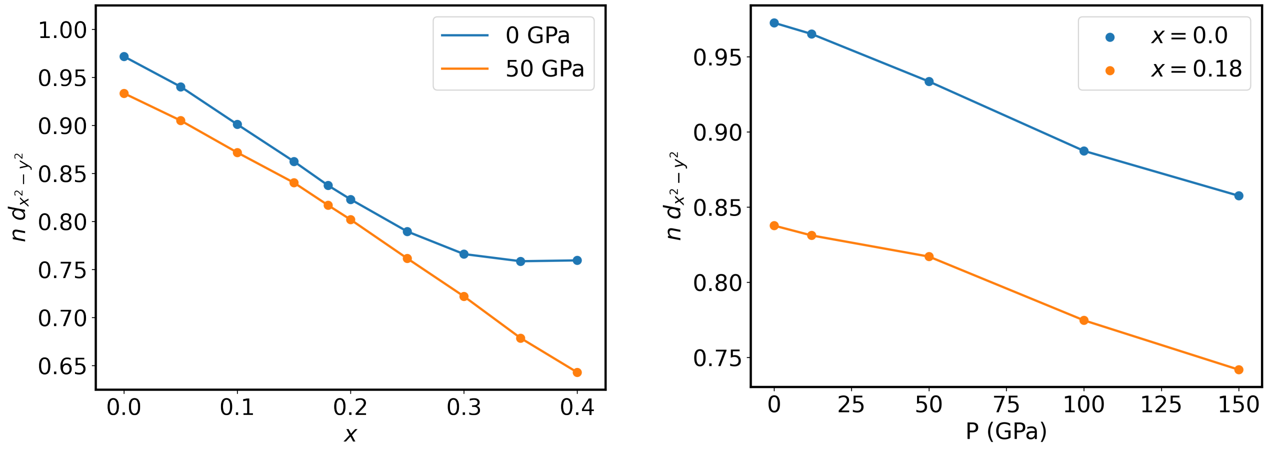

Figure S7: Left panel: Effective filling $n_{d_{x^2-y^2}}$ of the Ni-$d_{x^2-y^2}$ band as a function of Sr doping x, calculated in DMFT for the 10-band model. The results for 0 and 50 GPa are indicated as blue and orange lines, respectively. Right panel: $n_{d_{x^2-y^2}}$ of the Ni-$d_{x^2-y^2}$ band as a function of pressure at fixed doping $x = 0.00$ (blue curve) and $x = 0.18$ (orange curve). The hole doping of the main text is $\delta = 1 - n_{d_{x^2-y^2}}$.

In addition to Fig. 2 of the main text, we show in Fig S8 the DMFT bandstructure of the parent compound, PrNiO−2, at further pressures, visualizing the evolution with pressure. This is supplemented by the Fermi surface displayed in Fig. S9 for the same pressure and doping $x = 0$. Note that at 150 GPa the electron pocket around Γ becomes so large that it touches the Ni-$d_{x^2-y^2}$ band. At pressures higher than this point, the description of the correlated system in terms of 1 correlated orbital plus decoupled pockets likely needs to be refined.

In Fig. 3 of the main text, we showed the evolution of the 1-band DFT+DMFT spectrum of the parent compound ($x = 0$) as a function of pressure. This corresponds to path (c) in Fig. 1 and 4 of main text. Here, we also show the evolution with doping at 0 GPa [Fig. S10, path (a)] and 50 GPa [Fig. S11, path (b)]; as well as the pressure dependence for doping $x = 0.18$ [Fig. S12, path (d)].

To quantitatively assess the agreement between the 10- and 1-band model, in Tab. S4 we compare the effective mass for the two cases at zero physical doping ($x = 0$) as a function of pressure. The values are extracted directly from the imaginary part of the self-energy along the Matsubara axis, for vanishing imaginary frequency. The good agreement confirms the applicability of the 1-band model.

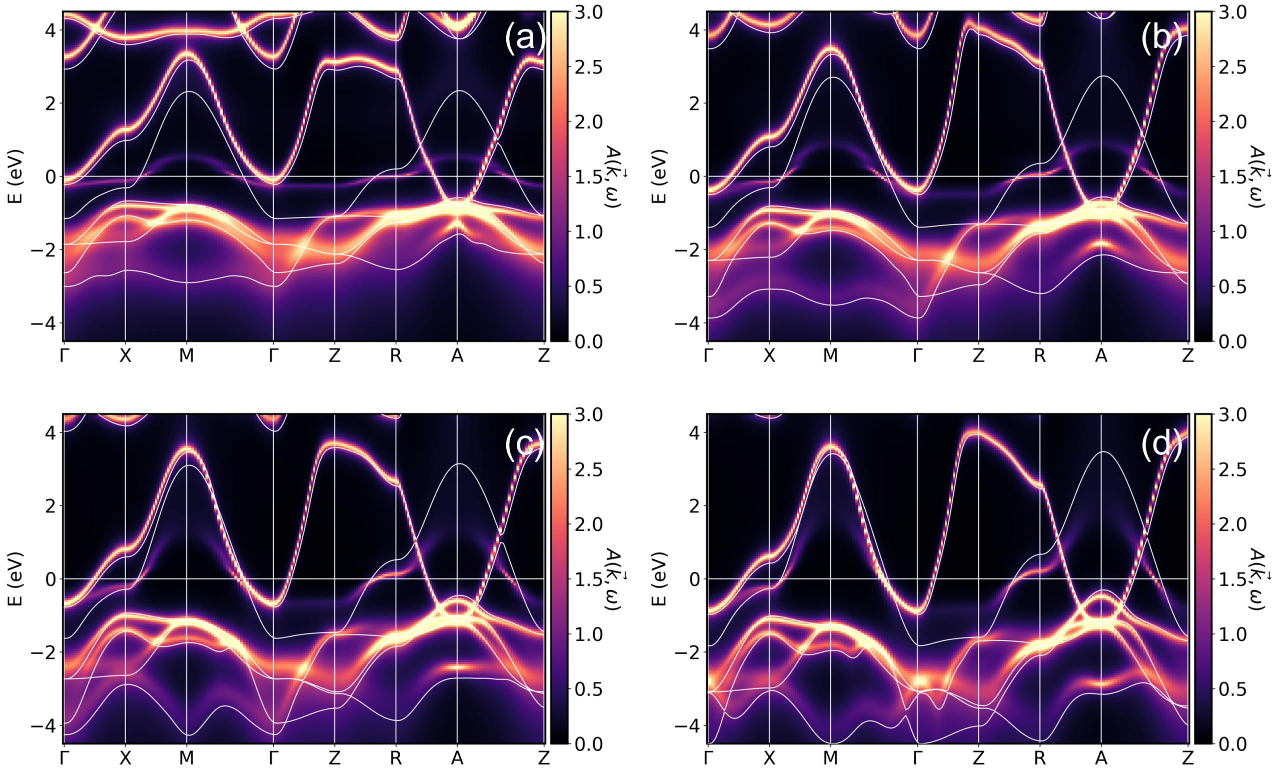

Figure S8: Evolution of the spectral function $A(\mathbf{k}, \omega)$ of $PrNiO_2$ along a high-symmetry path in reciprocal space for the 10-bands DMFT calculations. (a), (b), (c), and (d) correspond to calculations at 12, 50, 100, and 150 GPa, respectively. The spectral function $A(\mathbf{k}, \omega)$ is shown as a color scale. The DFT bands interpolated from Wannier functions are shown as white lines.

P [GPa]	m^*/m (10-bands)	m^*/m (1-band)
0	3.9	4.4
12	3.3	3.6
50	2.5	2.6
100	2.0	2.1
150	1.8	1.9

Table S4: Comparison of the effective mass m^*/m of the Ni $d_{x^2-y^2}$ band between the 10- and 1-band calculations at $x = 0$ as a function of pressure. The effective mass was computed as $m^*/m = 1 - \dfrac{\partial \mathrm{Im}\Sigma(i\omega_n \to 0)}{\partial i\omega_n}$.

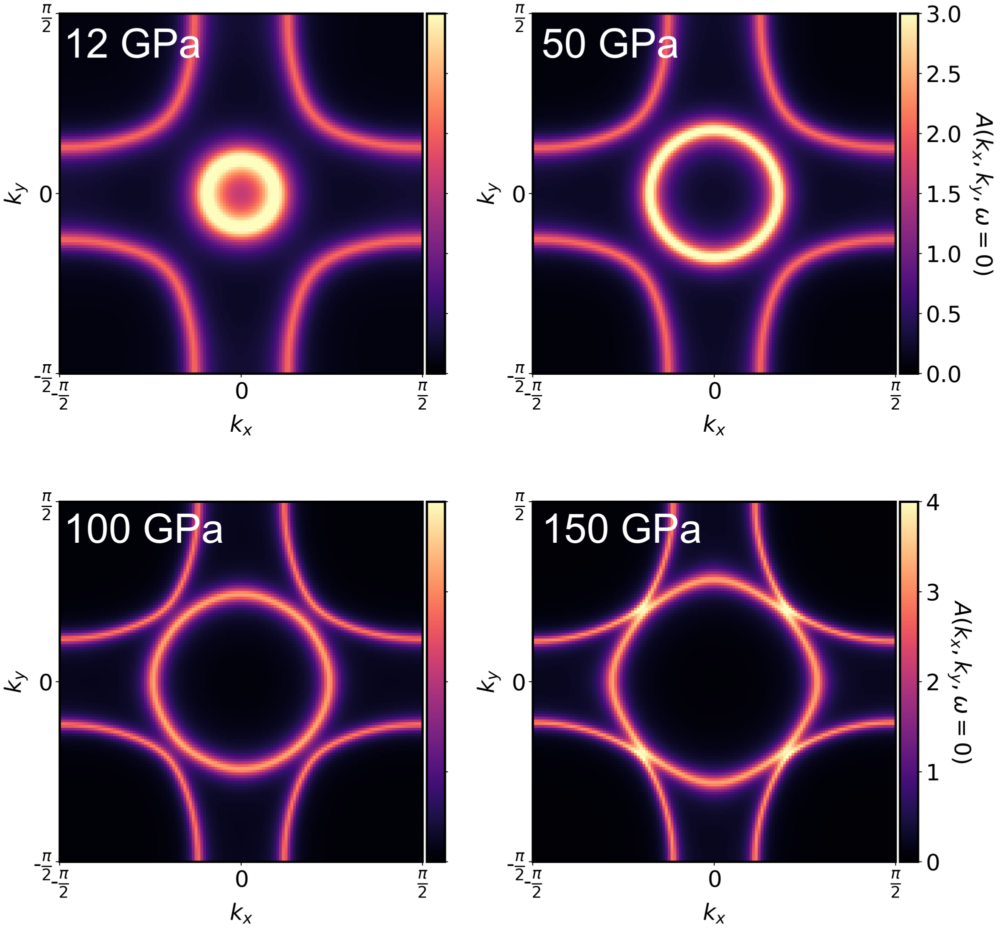

Figure S9: Evolution of the spectral function $A(\mathbf{k}, \omega)$ of $PrNiO_2$ along the Fermi surface at $k_z = 0$ for the undoped $PrNiO_2$ as a function of pressure.

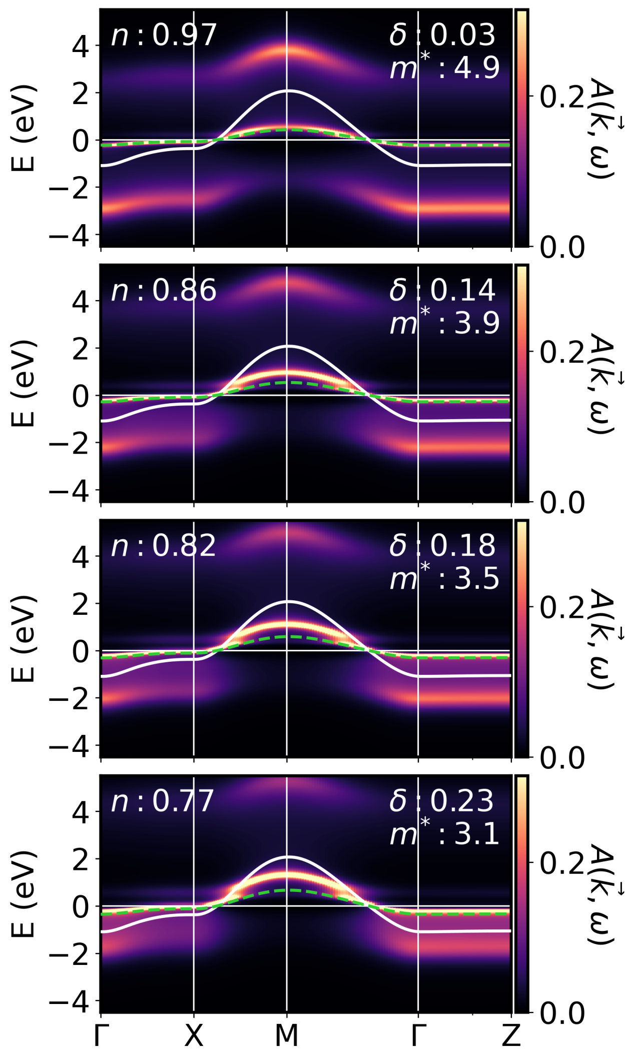

Figure S10: Spectral function computed from the local self-energy at fixed pressure P = 0 GPa as a function of doping x, from 0.0 to 0.30, at $\beta = 80t$. The spectral function $A(\mathbf{k}, \omega)$ is shown as a color gradient.

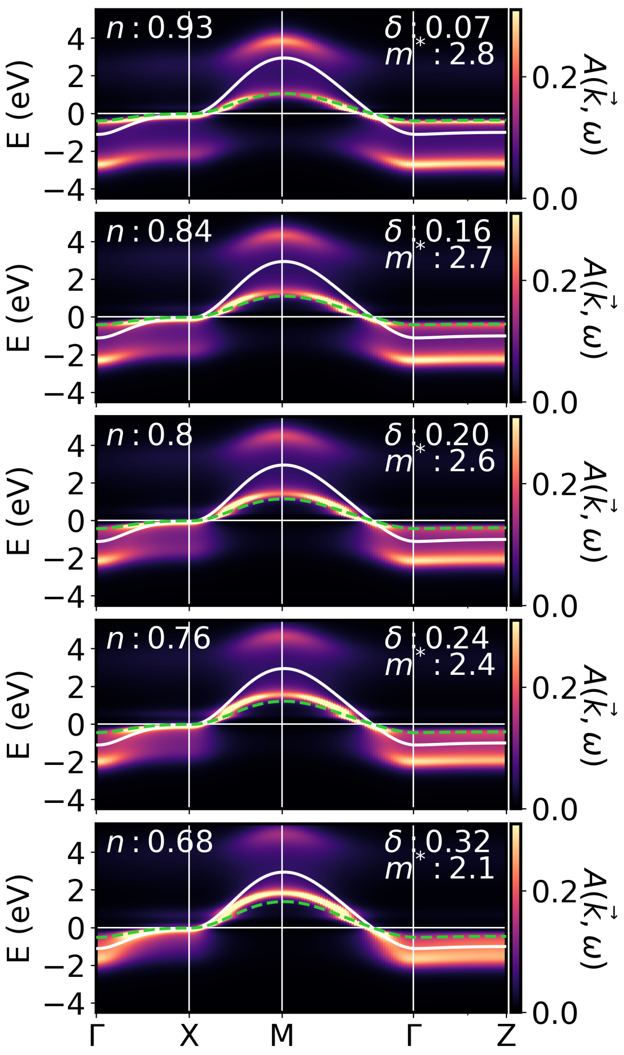

Figure S11: Spectral function computed from the local self-energy at fixed pressure P = 50 GPa as a function of doping x, from 0.0 to 0.30, at $\beta = 80t$. The spectral function $A(\mathbf{k}, \omega)$ is shown as a color gradient.

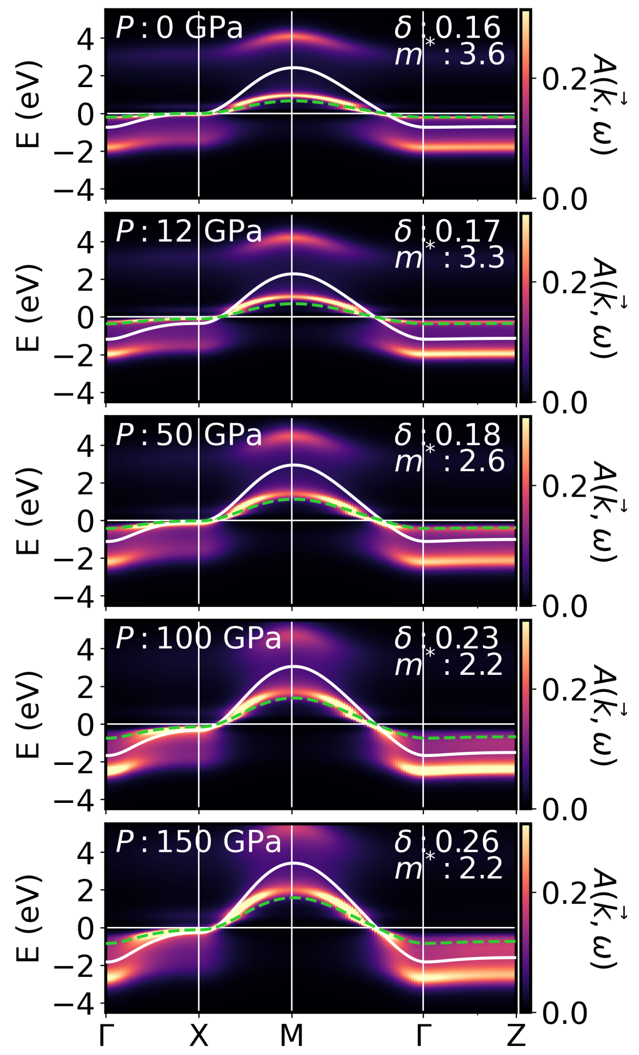

Figure S12: Spectral function computed from the local self-energy at fixed doping $x = 0.18$ and as a function of pressure, from 0 to 150, at $\beta = 80t$. The spectral function $A(\mathbf{k}, \omega)$ is shown as a color gradient.

B. Superconductivity in DΓA

In this section we present some additional comparisons of the superconducting phase diagram. First, in Fig. S13, we compare the superconducting dome of $Sr_xPr_{1-x}NiO_2$ to that of $Sr_xNd_{1-x}NiO_2$ calculated in [2]. Both phase diagrams are in good agreement and the deviations, including the slightly lower $T_c$in $Sr_xPr_{1-x}NiO_2$ can be explained by the different A cation (Pr instead of Nd).

Second in Fig. S14, we plot the change of T_c as a function of pressure together with the change of t. This comparison clearly reveals that the difference between the parent compound (left) and 18% Sr-doping (right) originates from the parent compound moving to optimal doping at 100 GPa, whereas the doped sample moves from optimal doping to overdoped with pressure.

Third, in Fig. S15 we provide additional information on how the superconducting T_c is obtained, namely by fitting the leading eigenvalue of the particle-particle ladder (Eliashberg equation) as a function of T. Comparing the left and right panel of Fig. S15 provides information on the accuracy of the fitting procedure. That is, the difference provides a crude estimate (rather overestimate) of the fitting error. Please also note that we are in the doped regime and hence there are no vertex divergences (poles in λ instead of crossings of $\lambda = 1$ [31]). The λ plotted is thus actually the leading eigenvalue and indicates a physical instability, i.e., a divergent superconducting susceptibility.

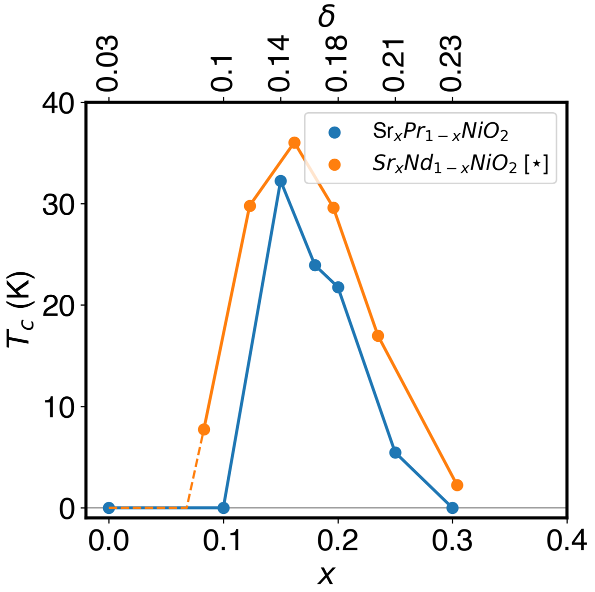

Figure S13: Comparison of the superconducting dome at 0 GPa calculated for $Sr_xPr_{1-x}NiO_2$ (our work) and for $Sr_xNd_{1-x}NiO_2$ (Ref. [2]).

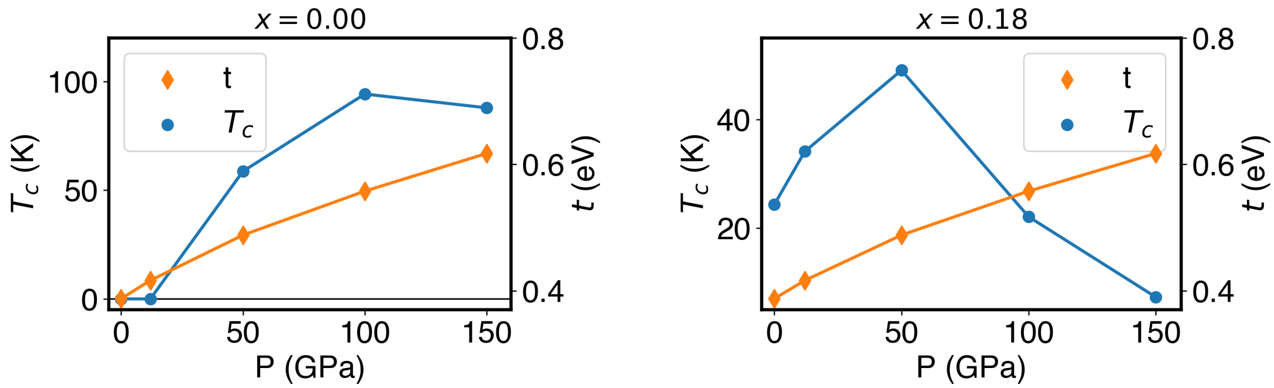

Figure S14: Trend of the superconducting T_c as a function of pressure, compared with the first-nearest-neighbor hopping t.

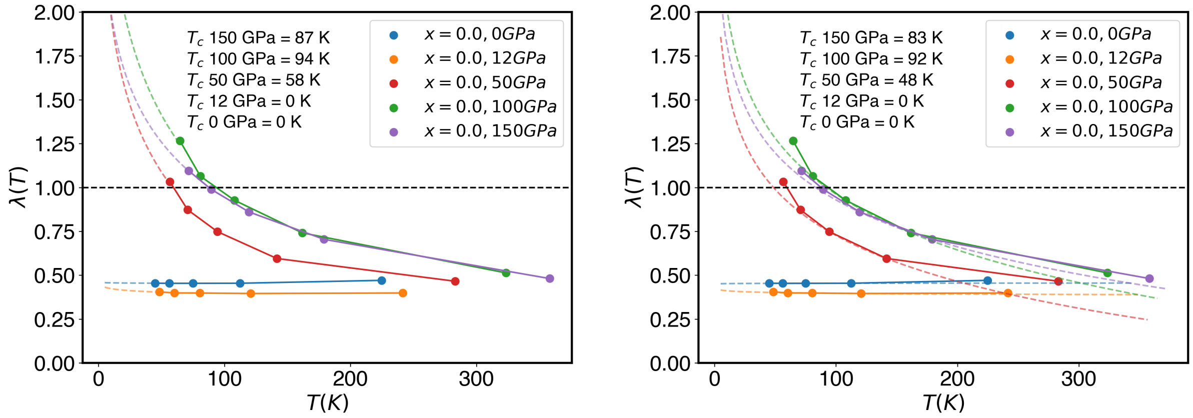

Figure S15: Leading superconducting eigenvalue λ as a function of temperature at $x = 0$ for different pressures. The critical temperature is the value at which the curve (or its extrapolation) crosses the $\lambda = 1$ line. Left: interpolant curve fitting using the last three points. Right: interpolant curve fitting excluding one point at the highest and two points at the lowest temperatures for the fit.

C. Tests of the Virtual Crystal Approximation

The Virtual Crystal Approximation should be approached carefully, especially when it is employed to interpolate between two atoms that are not adjacent on the periodic table.

We performed extensive checks of the quality of the Virtual Crystal Approximation (VCA) to simulate an average mixture of praseodymium (Pr) and strontium (Sr). In the following, we list the tests and their result, and briefly discuss their implication.

a. Vergard's Law The first simple test is to check that Vergard's Law is respected [40], i.e. that the lattice parameter of the intermediate alloy is a weighted mean of the two isolate compounds. In Fig. S16 we report a comparison between the lattice parameter of a Pr-Sr mixture in a face-centered cubic structure at different concentrations, obtained with the VCA and in a 2×2×2 supercell. We note that not only the law is respected, but even the small deviation from the linear behavior are matched by the calculations in the supercell, i.e. they are a physical deviation, rather than an artifact of the VCA.

b. c axis of $Pr_{0.75}Sr_{0.25}NiO_2$ We checked the consistency of the VCA in the specific environment of interest, i.e. the $Pr_{1-x}Sr_xNiO_2$ nickelate. A doping of $x = 0.25$ can be simulated in a relatively small 2×2×2 supercell, and its effect on the structural properties compared with the VCA result.

In Fig. S4 we show the change in enthalpy of $Pr_{0.75}Sr_{0.25}NiO_2$ as a function of the c axis using the VCA and in five different supercell configurations. The equilibrium c value thus obtained is identical for all the cases considered, and close to the experimental value [10], and deviations are only seen away from equilibrium.

c. Electronic structure The last test involves a direct comparison of the electronic band structure between the VCA case and the five inequivalent supercells at $x = 0.25$. We employed the relaxed structures describe in the previous paragraph, but

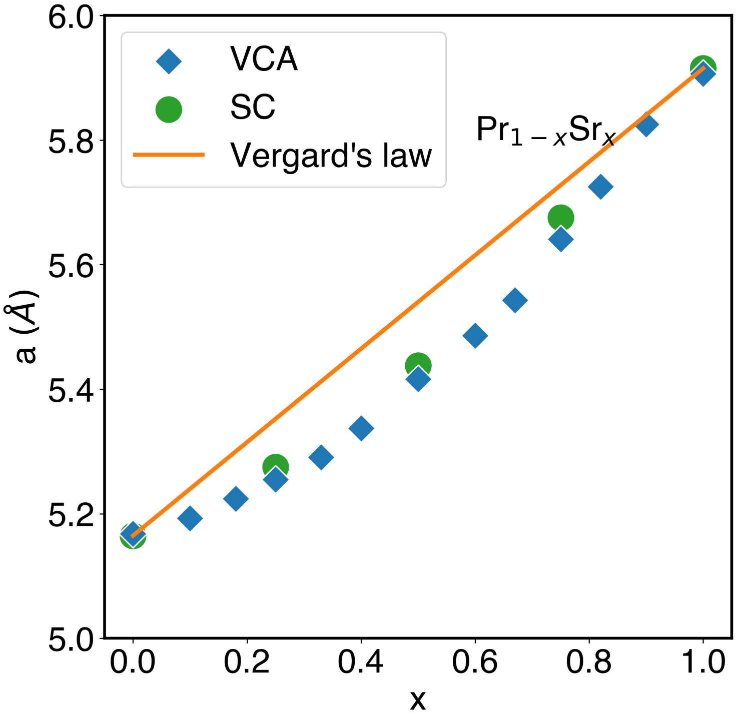

Figure S16: Vergard's Law for a Pr-Sr alloy in the face-centered cubic phase. Blue diamonds, green circles, and the orange line indicate the result obtained using the VCA, a 2×2×2 supercell, and the ideal law, respectively.

we note that their lattice parameters are identical within DFT accuracy. In Fig. S17 we show the electronic band structure for the VCA and the five different supercells. Also in this case the two results are in excellent agreement, with only a few minute differences due to the symmetry breaking induced by Sr in the supercell, which splits a few bands.

D. Further checks

In this section we include a few further checks and remarks concerning the necessary approximations employed in this work.

1. *Absence of self-consistency between DFT and DMFT*

The effect of local correlation, which changes the spectral function, may lead to a charge rearrangement that could be taken into account by a self-consistent DFT+DMFT scheme. In this work we did not employ such scheme. This is mainly justified by the result shown in Fig. S18, where we show a comparison between the spectral function of the relevant, correlated orbital of Ni $d_{x^2-y^2}$ (solid red line) and its corresponding Wannier function (black dashed lines). For this orbital we obtain an occupation of 0.41 in the uncorrelated case and 0.49 in the correlated one. The occupation of all the other orbitals changes even less.

2. *Possible temperature dependence of the occupation of the Ni $d_{x^2-y^2}$ band*

The 10-band calculations employed to determine the occupation of the Ni $d_{x^2-y^2}$ band (See Fig. S1), were all performed at a fixed temperature of 300 K, as it is below the quasiparticle bandwidth, and we did not expect differences on the DMFT level. In principle, a temperature dependence may be in effect if temperature changed significantly the occupation of the Ni $d_{x^2-y^2}$ orbital, leading to a quantitative change in its superconducting properties.

In Figs. S18 and S19, we show a comparison of results computed at 100 and 300 K, at 50 GPa and in absence of doping. In particular, we compare the spectral function and self energy, respectively. Both are almost the same, and the occupation of the Ni $d_{x^2-y^2}$ orbital changes by less than 10^{-4} per Ni. This confirms that temperature variations do not impact the DMFT results.

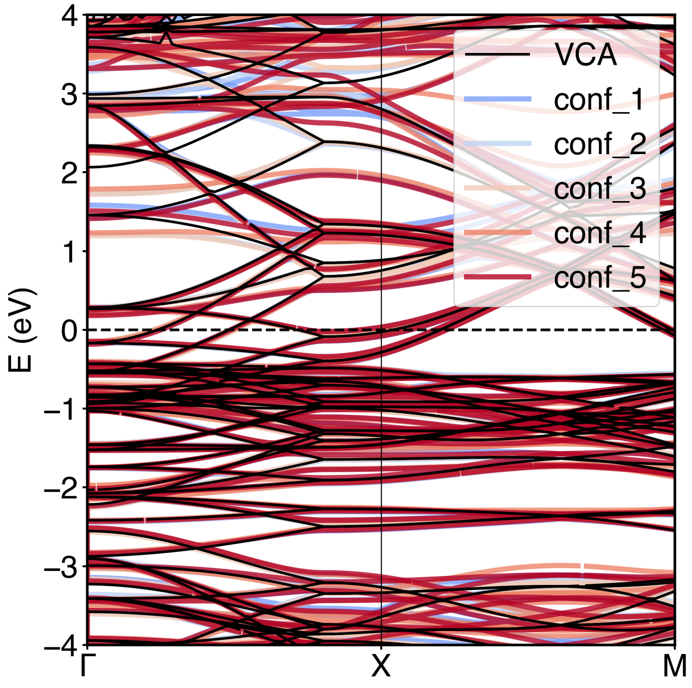

Figure S17: Electronic band structure for $Pr_{0.75}Sr_{0.25}NiO_2$ using the VCA and in five different supercells. The VCA value is shown as a black line, and the results for the supercells are shown as colored lines.

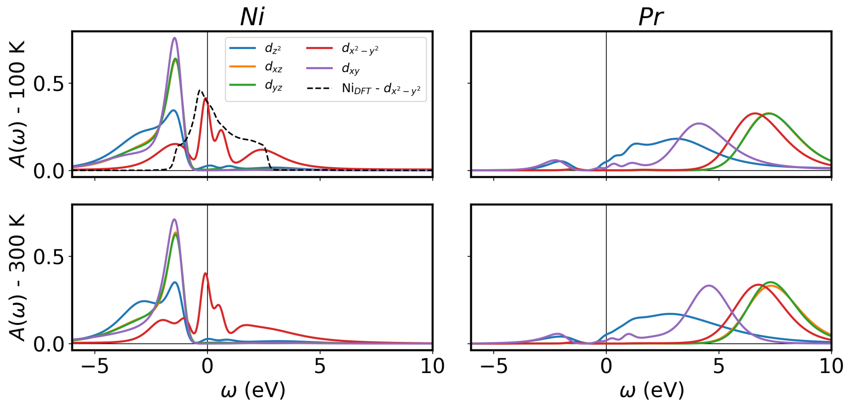

Figure S18: Analytically continued local Green's function for the five orbitals of Ni (left) and Pr (right) in undoped $PrNiO_2$ at 50 GPa, 100 K (top) and 300 K (bottom). The correlated spectral functions for the d_{z^2}, d_{xz}, d_{yz}, $d_{x^2-y^2}$, and d_{xy} orbitals are shown as solid blue, orange, green, red and purple lines, respectively. The uncorrelated, DFT DOS for Ni $d_{x^2-y^2}$ is shown as a black dashed line (top left panel).

3. *Competition with antiferromagnetism*

Cluster DMFT studies [41] have shown that in nickelates antiferromagnetic (AFM) order can compete with superconductivity for small dopings at zero pressure. The λ correction employed in our DΓA calculation suppresses antiferromagnetism completely, fulfilling the Mermin-Wagner theorem. To estimate the role that competition with AFM order might have, we computed the inverse DMFT antiferromagnetic susceptibility (i.e., solve the Bethe-Salpeter equation without λ correction). Here we focus on the undoped parent compound ($x = 0$) at constant β (approximately 200 K). The result is shown in Fig. S20. Negative susceptibilities in the (enforced) paramagnetic phase signal AFM in DMFT. At a pressure of about 130 GPa the pressure

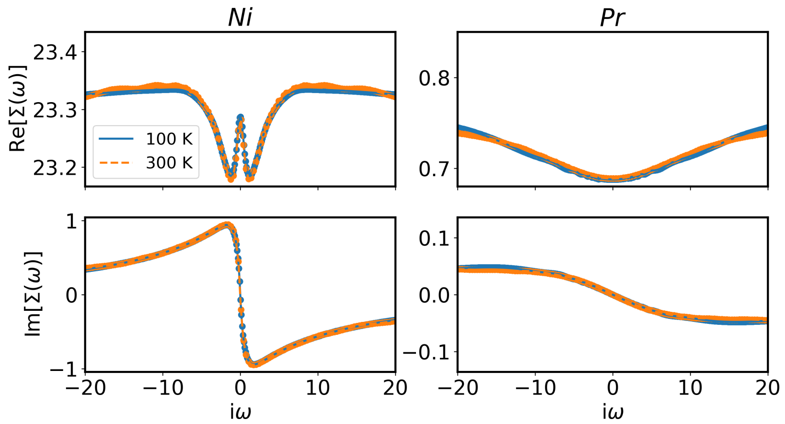

Figure S19: Local selfenergy for the Ni and Pr $d_{x^2-y^2}$ orbital at 100 and 300 K (solid blue and dashed orange lines, respectively) and 50 GPa. **Note:** the data were plotted skipping one in two data points for visual clarity.

induced-doping is sufficient to suppress AFM even in DMFT. Please note that DMFT largely overestimates antiferromagnetism for (essentially) two-dimensional nickelates, as AFM is suppressed by non-local fluctuatons. We expect the actual antiferromagnetism to be somewhere in-between the DΓA result ($T_N = 0$) and the (2×2) cluster DMFT [41] if a weak coupling in the third dimension and/or the finiteness of the experimental film is taken into account and leads to a finite Néel temperature (T_N).

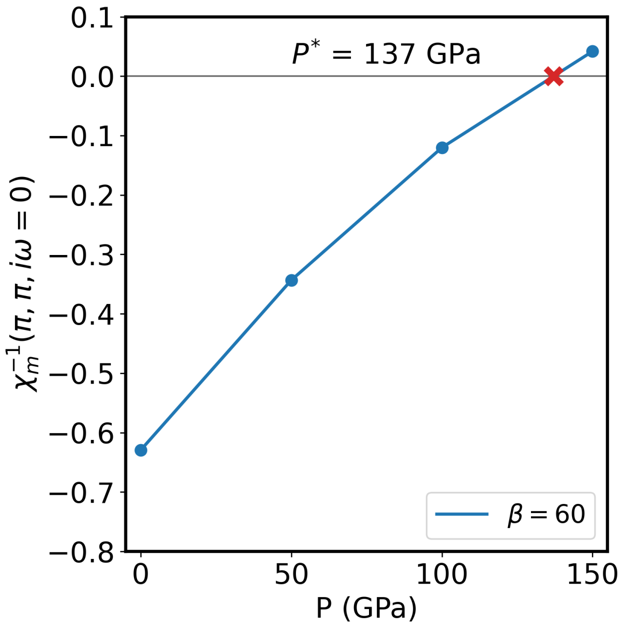

Figure S20: Inverse antiferromagnetic DMFT susceptibility $\chi_m(\pi, \pi, i\omega = 0)^{-1}$ as a function of pressure for $LaNiO_2$ at $\beta = 60$ in units of t (with t as reported in Tab. 1 of the main text). The pressure at which the AFM instability disappears even in DMFT is shown as a red x.

[1] Additional data related to this publication, including input/output files and raw data for both DFT and DMFT calculations is available at DOI:10.48436/9xych-d8n28.

[2] M. Kitatani, L. Si, O. Janson, R. Arita, Z. Zhong, and K. Held, npj Quantum Materials **5**, 59 (2020).

[3] P. Worm, Dgapy (2023).
[4] G. Kresse and J. Furthmüller, Phys. Rev. B **54**, 11169 (1996).
[5] J. P. Perdew, A. Ruzsinszky, G. I. Csonka, O. A. Vydrov, G. E. Scuseria, L. A. Constantin, X. Zhou, and K. Burke, Phys. Rev. Lett. **100**, 136406 (2008).
[6] G. Kresse and J. Furthmüller, Phys. Rev. B **59**, 1758 (1999).
[7] L. Bellaiche and D. Vanderbilt, Phys. Rev. B **61**, 7877 (2000).
[8] V. Christiansson, F. Petocchi, and P. Werner, Phys. Rev. B **107**, 045144 (2023).
[9] D. Li, K. Lee, B. Y. Wang, M. Osada, S. Crossley, H. R. Lee, Y. Cui, Y. Hikita, and H. Y. Hwang, Nature **572**, 624 (2019).
[10] M. Osada, B. Y. Wang, B. H. Goodge, K. Lee, H. Yoon, K. Sakuma, D. Li, M. Miura, L. F. Kourkoutis, and H. Y. Hwang, Nano Letters **20**, 5735 (2020).
[11] S. Zeng, C. S. Tang, X. Yin, C. Li, M. Li, Z. Huang, J. Hu, W. Liu, G. J. Omar, H. Jani, Z. S. Lim, K. Han, D. Wan, P. Yang, S. J. Pennycook, A. T. S. Wee, and A. Ariando, Phys. Rev. Lett. **125**, 147003 (2020).
[12] K. Lee, B. H. Goodge, D. Li, M. Osada, B. Y. Wang, Y. Cui, L. F. Kourkoutis, and H. Y. Hwang, APL Materials **8**, 041107 (2020).
[13] M. Osada, B. Y. Wang, B. H. Goodge, S. P. Harvey, K. Lee, D. Li, L. F. Kourkoutis, and H. Y. Hwang, Advanced Materials **n/a**, 2104083 (2021).
[14] N. N. Wang, M. W. Yang, K. Y. Chen, Z. Yang, H. Zhang, Z. H. Zhu, Y. Uwatoko, X. L. Dong, K. J. Jin, J. P. Sun, and J. G. Cheng, Nature Communications **13**, 4367 (2022).
[15] A. Ikeda, T. Manabe, and M. Naito, Physica C **506**, 83 (2014).
[16] K. Lee, B. Y. Wang, M. Osada, B. H. Goodge, T. C. Wang, Y. Lee, S. Harvey, W. J. Kim, Y. Yu, C. Murthy, *et al.*, arXiv preprint arXiv:2203.02580 (2022).
[17] S. Zeng, C. Li, L. E. Chow, Y. Cao, Z. Zhang, C. S. Tang, X. Yin, Z. S. Lim, J. Hu, P. Yang, *et al.*, Science advances **8**, eabl9927 (2022).
[18] F. D. Murnaghan, Proceedings of the National Academy of Sciences of the United States of America **30**, 244 (1944), http://www.pnas.org/content/30/9/244.full.pdf+html.
[19] A. A. Mostofi, J. R. Yates, Y.-S. Lee, I. Souza, D. Vanderbilt, and N. Marzari, Computer Physics Communications **178**, 685 (2008).
[20] T. Miyake and F. Aryasetiawan, Phys. Rev. B **77**, 085122 (2008).
[21] T. Miyake, F. Aryasetiawan, and M. Imada, Phys. Rev. B **80**, 155134 (2009).
[22] M. Methfessel, M. van Schilfgaarde, and R. Casali, in Electronic Structure and Physical Properties of Solids: The Uses of the LMTO Method, Lecture Notes in Physics. H. Dreysse, ed. **535**, 114 (2000).
[23] J. M. Tomczak, T. Miyake, R. Sakuma, and F. Aryasetiawan, Phys. Rev. B **79**, 235133 (2009).
[24] O. Ivashko, M. Horio, W. Wan, N. Christensen, D. McNally, E. Paris, Y. Tseng, N. E. Shaik, H. M. Rønnow, H. I. Wei, C. Adamo, C. Lichtensteiger, M. Gibert, M. R. Beasley, K. M. Shen, J. M. Tomczak, T. Schmitt, and J. Chang, Nature Comm. **10**, 786 (2019).
[25] L. Si, W. Xiao, J. Kaufmann, J. M. Tomczak, Y. Lu, Z. Zhong, and K. Held, Phys. Rev. Lett. **124**, 166402 (2020).
[26] L. Vaugier, H. Jiang, and S. Biermann, Phys. Rev. B **86**, 165105 (2012).
[27] A. Georges, G. Kotliar, W. Krauth, and M. J. Rozenberg, Rev. Mod. Phys. **68**, 13 (1996).
[28] K. Held, Advances in physics **56**, 829 (2007).
[29] N. Parragh, A. Toschi, K. Held, and G. Sangiovanni, Phys. Rev. B **86**, 155158 (2012).
[30] M. Wallerberger, A. Hausoel, P. Gunacker, A. Kowalski, N. Parragh, F. Goth, K. Held, and G. Sangiovanni, Comp. Phys. Comm. **235**, 388 (2019).
[31] M. Kitatani, R. Arita, T. Schäfer, and K. Held, Journal of Physics: Materials **5**, 034005 (2022).
[32] A. Toschi, A. A. Katanin, and K. Held, Phys Rev. B **75**, 045118 (2007).
[33] A. A. Katanin, A. Toschi, and K. Held, Phys. Rev. B **80**, 075104 (2009).
[34] G. Rohringer, A. Katanin, T. Schäfer, A. Hausoel, K. Held, and A. Toschi, github.com/ladderDGA (2018), github.com/ladderDGA.
[35] P. Worm, *Dynamical vertex approximation for correlated electron systems*, Ph.D. thesis, TU Wien (2021).
[36] T. Schäfer, N. Wentzell, F. Šimkovic, Y.-Y. He, C. Hille, M. Klett, C. J. Eckhardt, B. Arzhang, V. Harkov, F. m. c.-M. Le Régent, A. Kirsch, Y. Wang, A. J. Kim, E. Kozik, E. A. Stepanov, A. Kauch, S. Andergassen, P. Hansmann, D. Rohe, Y. M. Vilk, J. P. F. LeBlanc, S. Zhang, A.-M. S. Tremblay, M. Ferrero, O. Parcollet, and A. Georges, Phys. Rev. X **11**, 011058 (2021).
[37] J. Kaufmann, C. Eckhardt, M. Pickem, M. Kitatani, A. Kauch, and K. Held, Phys. Rev. B **103**, 035120 (2021).
[38] M. Kitatani, T. Schäfer, H. Aoki, and K. Held, Phys. Rev. B **99**, 041115 (2019).
[39] G. Li, A. Kauch, P. Pudleiner, and K. Held, Comp. Phys. Comm. **241**, 146 (2019).
[40] A. R. Denton and N. W. Ashcroft, Physical Review A **43**, 3161 (2004).
[41] J. Karp, A. Hampel, and A. J. Millis, Phys. Rev. B **105**, 205131 (2022).
